# An Exploration of the Antioxidative and Anti-Inflammatory Role of *Lactiplantibacillus plantarum* 106 via Improving Mitochondrial Function

**DOI:** 10.3390/foods13131981

**Published:** 2024-06-24

**Authors:** Mengchun Qin, Yinfei Xing, Maocheng Sun, Lin Ma, Xiaolei Li, Fumin Ma, Dan Li, Cuicui Duan

**Affiliations:** 1 Key Laboratory of Agro-Products Processing Technology, Education Department of Jilin Province, Changchun University, 6543 Weixing Road, Changchun 130022, China; 2 College of Veterinary Medicine, Jilin University, Changchun 130062, China; xingyf23@mails.jlu.edu.cn

**Keywords:** *Lactiplantibacillus plantarum*, antioxidant, bioinformatics analysis, mitochondrial function, apoptosis

## Abstract

In this present study, bioinformatics analysis and the experimental validation method were used to systematically explore the antioxidant activity and anti-inflammatory effect of *Lactiplantibacillus plantarum* A106, which was isolated from traditional Chinese pickles, on lipopolysaccharide (LPS)-induced RAW264.7 macrophages. *L. plantarum* A106 had a good scavenging ability for DPPH, ABTS, and hydroxyl radicals. Furthermore, *L. plantarum* A106 could increase the activity of RAW264.7 macrophages; raise the SOD and GSH levels, with or without LPS sensitization; or decrease the MDA, TNF–α, and IL–6 levels. In order to deeply seek the antioxidant and anti-inflammatory role and mechanism, bioinformatic analysis, including GO, KEGG, and GSEA analysis, was used to conduct an in-depth analysis, and the results showed that the LPS treatment of RAW264.7 macrophages significantly upregulated inflammatory-related genes and revealed an enrichment in the inflammatory signaling pathways. Additionally, a network analysis via the Cytoscape software (version 3.9.1) identified key central genes and found that LPS also disturbed apoptosis and mitochondrial function. Based on the above bioinformatics analysis, the effects of *L. plantarum* A106 on inflammation-related gene expression, mitochondrial function, apoptosis, etc., were detected. The results indicated that *L. plantarum* A106 restored the declined expression levels of crucial genes like TNF–α and IL–6; mitochondrial membrane potential; and apoptosis and the expression of apoptosis-related genes, Bcl–2, Caspase–3, and Bax. These results suggest that *L. plantarum* A106 exerts antioxidant activity and anti-inflammatory effects through regulating inflammatory and apoptosis-related gene expression, restoring the mitochondrial membrane potential.

## 1. Introduction

Lactic acid bacteria (LAB), a class of probiotics, not only maintain the ecological balance of the intestinal microbiota but also exhibit biologically active properties that have antitumor, immunomodulatory, and antioxidant effects. These properties have contributed to extensive research and application in the food and health product industries. Recently, LAB have been widely recognized for their crucial role in modulating inflammatory responses, including the regulation of cytokine production, the improvement of the intestinal barrier function, and the increase of immune cell activity [1,2]. In one study, *L. reuteri* LM1071 inhibited the production of inflammatory mediators like NO, pro-inflammatory factors, and COX proteins, thus demonstrating anti-inflammatory potential [3]. In another, *L. plantarum* DP189 downregulated pro-inflammatory cytokines such as TNF–α, IL–6, and IL–1β in neurodegenerative diseases, thereby inhibiting inflammatory responses and modulating the gut microbiota [4]. Another study, which used a D-galactose-induced senescent mouse model to explore the antioxidant effects of lactobacillus on hepatotoxicity, found that *L. plantarum* AR113 and AR501 attenuated liver injury and reduced the abnormal activities of SOD, GSH-Px, and catalase to normal levels in mice [5]. Oxidative stress was generated during the inflammatory response process, which could reduce the cellular immune function and increase and aggregate the inflammatory factors in cells, thereby promoting cell damage. Consequently, the level of inflammatory factors in the cells was a direct indication of the level of oxidative stress in the cells [6].

The RAW264.7 cell model, derived from murine macrophages, has been extensively utilized to study immune responses, particularly in the context of inflammation and infection. Lipopolysaccharide (LPS)-treated RAW264.7 macrophages were commonly used to explore the immune response mechanisms. It was reported that LPS could activate macrophages and induce the secretion of several pro-inflammatory cytokines, including tumor necrosis factor-alpha (TNF–α), interleukin–1β (IL–1β), IL–6, etc. [7,8]. A study investigated the anti-inflammatory potential and underlying mechanisms of andrographolide in LPS-induced RAW264.7 macrophages, revealing that andrographolide inhibited the activation of NF-κB and MAPK, thereby inhibiting autophagy and alleviating the inflammatory response [9]. Additionally, the phenotype and function of the macrophages were regulated by their surrounding microenvironment, and they were susceptible to reactive oxygen species (ROS), leading to cell death [10]. Thus, macrophages can also serve as a cellular model for oxidative stress. Under exogenous stimuli, macrophages could phagocytose, process, and present foreign substances, a process accompanied by electron transfer, which was a primary source of oxidative stress [11]. Tang et al. (2019) found that both quercetin and quercitrin could suppress the LPS-induced inflammatory response and oxidative stress in macrophages [12]. *L. paracasei* KW3110 inhibited the mitochondrial dysfunction induced by LPS in RAW264.7 macrophages, which, in turn, alleviated inflammatory stress [13]. However, the potential targets, signaling pathways, organelles, etc. still needed deep analysis before the anti-inflammatory and antioxidative mechanisms could be explored. Bioinformatics analysis provided a comprehensive and precise research direction. For example, some crucial bioactive ingredients, signaling pathways, and potential targets have been acquired via bioinformatics analysis, including the Gene Ontology (GO) and Kyoto Encyclopedia of Genes and Genomes (KEGG) analyses, as well as accounts of protein–protein interaction (PPI) [14]. However, there are few reports on how LAB modulate the LPS-induced inflammatory process and oxidative stress response in RAW264.7 macrophages using bioinformatics analysis combined with in vitro validation.

This study employed bioinformatics analysis and experimental validation methods to investigate the role of *L. plantarum* A106 in modulating inflammatory processes and relieving oxidative stress responses utilizing the LPS-induced oxidative stress in RAW264.7 macrophages model. Specifically, this study explored the protective effects of *L. plantarum* A106 against LPS-induced inflammation and oxidative stress in RAW264.7 macrophages. This study will enhance our understanding of the anti-inflammatory and antioxidative mechanisms of probiotics and may provide a scientific basis for developing new strategies to treat inflammation and oxidative stress-related diseases.

## 2. Materials and Methods

### 2.1. Strain and Cell Line

*L. plantarum* A106 was isolated from the naturally fermented pickles bought from the Yanbian area of Jilin Province. It was cultured in a De Man–Rogosa–Sharp (MRS) broth. The RAW264.7 macrophages were gifted by Jilin University.

### 2.2. Reagents and Instruments

1,1-Diphenyl-2-trinitrophenylhydrazine (DPPH) and 2,2′-Azido-bis-(3-ethylbenzodihydrothiazoline-6-sulfonic acid) (ABTS) were purchased from Phygene Life Sciences Co., Ltd. (Fuzhou, China). The DEME/high glucose medium was purchased from HyClone (HyClone Laboratories, Inc., Omaha, NE, USA). The serum was bought from Inner Mongolia OPCEL Biotechnology Co., Ltd. (Inner Mongolia, China). The cell counting kit-8 (CCK-8), superoxide dismutase (SOD), reactive oxygen species (ROS), mitochondrial membrane potential, and apoptosis kits were purchased from Beyotime Biotechnology (Beijing, China).

### 2.3. Culture of L. plantarum A106 and Preparation of Different Components

The *L. plantarum* A106 that was stored at −80 °C was quickly dissolved in a 37 °C water bath and inoculated into the MRS liquid medium with a 3% inoculum. It was incubated at 37 °C for 24 h and subsequently subcultured to 2–3 generations for further experiments.

With respect to the preparation of the culture supernatant and bacterial cells, the *L. plantarum* A106 was put in a shaking incubator (37 °C, 150 rpm) for 18–24 h, then a centrifuge at 5610× *g* for 20 min at 4 °C, before the supernatant was collected and filtered through a 0.22 µm filter membrane. The bacterial cells were washed three times with PBS (0.01 mol/L at a pH of 7.4) and adjusted to 1 × 10^9^ CFU/mL.

With respect to the preparation of the cell-free extract, the lysozyme (1.5 mg/mL) was added to intact *L. plantarum* A106 with a concentration of 1 × 10^9^ CFU/mL, then put in a water bath at 37 °C for 30 min, accompanied by an ultrasonic cell crusher in an ice bath (250 W, 5 s of operation, 10 s of interval, and a duration of 30 min). Afterward, the mixture was centrifuged at 5610× *g*/min for 10 min to remove any bacterial debris and to obtain the cell-free extracts.

### 2.4. Effectiveness of Different Fractions of L. plantarum A106 on Free Radical Scavenging

#### 2.4.1. DPPH Scavenging Rate

The DPPH scavenging rate was tested according to the method reported by Xu et al. (2019), making appropriate modifications [15]. Specifically, 1 mg of DPPH was dissolved in 25 mL of anhydrous ethanol. Subsequently, 500 µL of different components were thoroughly mixed with 500 µL of DPPH-anhydrous ethanol solution. There was centrifugation at 4909× *g* for 10 min, before the supernatant was discarded, and the absorbance was tested at 517 nm. The DPPH scavenging rate was calculated according to the following formula:

DPPH clearance (%) = [1 − (*A*_2_ − *A*_1_)/*A*_0_] × 100

where *A*_0_ is the absorbance of the ultrapure water and DPPH; *A*_1_ is the absorbance of the sample and ultrapure water; and *A*_2_ is the absorbance of the sample and DPPH.

#### 2.4.2. ABTS^+^ Scavenging Rate

The ABTS^+^ scavenging rate was tested according to the method reported by Li et al. (2015), with minor modifications [16]. The ABTS reaction solution was prepared according to the instructions. In total, 300 µL of various fractions were separately mixed with 300 µL of the ABTS reaction solution. The mixture was shook for 30 s and then kept in dark conditions for 6 min, followed by centrifuging at 5610× *g* for 5 min. The supernatant was transferred to a 96-well plate to read at 734 nm. The ABTS^+^ scavenging rate was calculated according to the following formula:

ABTS^+^ scavenging rate (%) = (1 − *A*_1_/*A*_0_) × 100

where *A*_0_ is the absorbance of the ABTS working solution and *A*_1_ is the absorbance of the sample.

#### 2.4.3. Hydroxyl Radical Scavenging Rate

The hydroxyl radical scavenging rate was detected according to the method described by Yang et al. (2017), with appropriate modifications [17]. Firstly, 0.5 mL of 1,10-o-diazophenanthrene (1.875 mmol/L), 1 mL of PBS (0.01 mmol/L at a pH of 7.4), and 0.5 mL of ferrous sulphate (1.875 mmol/L) were added to a 5 mL Eppendorf tube and mixed thoroughly. Then, 0.5 mL of the samples and 0.5 mL of H_2_O_2_ (0.1%) were added, and the mixture was vortexed thoroughly and incubated in a water bath at 37 °C for 1 h. Following this, the samples were centrifuged for 10 min at 5610× *g* and the supernatant was transferred to a 96-well plate to read at 536 nm. The hydroxyl radical scavenging rate was calculated according to the following formula:

Hydroxyl radical scavenging rate (%) = [(*A_s_* − *A_n_*)/(*A_b_* − *A_n_*)] × 100

where *A_s_* is the absorbance of the samples and H_2_O_2_; *A_n_* is the absorbance of the PBS; and *A_b_* is the absorbance of the H_2_O_2_.

### 2.5. Cell Viability Assay

The RAW264.6 macrophages were cultivated in a cell culture incubator at 37 °C with 5% CO_2_ using the DMEM/high glucose medium supplemented with 10% fetal bovine serum. After subculturing for 2–3 generations, the cells were digested with trypsin, centrifuged, resuspended in the complete medium, and adjusted to a concentration of 1 × 10^6^ cells/mL, before being inoculated in a 96-well plate with a 200 µL cell suspension per well and cultured for 24 h. In the experimental group, 50 µL of bacterial suspension was added to each well at a different ratio: 1:100 (1 × 10^4^ CFU/mL) or 1:1000 (1 × 10^3^ CFU/mL) relative to the cell concentration using the complete medium. The control group only used the complete medium. Afterwards, 1 µg/mL of LPS was added to the experimental group 4 h before the end of the cultivation; meanwhile, 20 µL of CCK-8 solution was added to each well. The cells were then incubated at 37 °C for 1.5, 2, 2.5, and 3 h. The absorbance values at 450 nm were measured at various incubation periods. The cell viability was determined using the following formula:

Cell viability = [(*A*_2_ − *A*_0_)/(*A*_1_ − *A*_0_)] × 100%

where *A*_0_ is the absorbance of the positive group, *A*_1_ is the absorbance of the control group, and *A_2_* is the absorbance of the experimental group.

### 2.6. Measurement of the Levels of the Cytokines, TNF–α and IL–4

The cells were harvested and centrifuged, and the culture supernatant was collected. The cytokines TNF–α and IL–4 in the supernatant of the culture that underwent different treatments were detected using commercial ELISA kits (BioLegend, San Diego, CA, USA) according to the manufacturer’s instruction.

### 2.7. Download of Microarray Datasets and Extraction of Differentially Expressed Genes (DEGs) in Microarray Datasets

The dataset GSE186886, downloaded from the GEO database (http://www.ncbi.nlm.nih.gov/geo/), included the control group and LPS-induced RAW264.7 group.

The “limma” (version 3.58.1) R package was employed to identify the differentially expressed genes 1 (DEGs1) between the LPS-induced group and the control group in the GSE186886 dataset, where *p* < 0.05 and |log2 (fold change, FC)| > 1 were set as thresholds. Subsequently, “ggplot2” (version 3.5.0) was utilized to construct a volcano plot illustrating the expression of the DEGs.

### 2.8. Functional Enrichment Analysis

Both the Gene Ontology (GO) and Kyoto Encyclopedia of Genes and Genomes (KEGG) pathways analysis of the DEGs2 (differentially expressed genes in DEGs1 with |log2 (fold change, FC)| > 2) were conducted using the DAVID (https://davidbioinformatics.nih.gov/) online platform, with *p*-values < 0.05 and q-values < 0. The results were visualized using the “ggplot2” R package.

In our study, we conducted an enrichment analysis of the DEGs2 targeting the KEGG pathways, GO terms, and Reactome pathways using the “clusterProfiler” (version 4.10.1) in R. The gene set enrichment analysis (GSEA) method was employed to associate the DEGs2 with KEGG pathways, GO terms, and Reactome pathways, considering *p* < 0.05 as significant. For the visualization of these enrichment outcomes, the “ggplot2” package in R was utilized.

### 2.9. Protein–Protein Interaction (PPI) Network Analysis

To elucidate the functional interactions among the screened potential proteins, the identified DEGs2 were imported into the STRING database (https://string-db.org/) to construct a protein–protein interaction (PPI) network, where the confidence score threshold was set to 0. Subsequently, the PPI network was imported into the Cytoscape software (version 3.9.1), where the significant modules were depicted using the CytoNCA (version 2.1.6) and CytoHubba (version 0.1) plugin.

### 2.10. Gene Ontology and Pathway Enrichment Analysis Using Metascape

A Metascape analysis offers the advantage of integrating multiple gene list analysis and enrichment tools within a single platform, thereby providing comprehensive, interactive visualizations for a deeper understanding of biological functions and pathways. To deeply explore the biological functions and pathways associated with the genes related to the mitochondrial pathways identified in the Gene Ontology Cellular Component (GO CC) analysis, an enrichment analysis was conducted using Metascape (https://metascape.org). Finally, Metascape’s interactive visualization tools were used to present the results.

### 2.11. Quantitative Real-Time Polymerase Chain Reaction (qPCR)

The total RNA was extracted using Trizol (Sigma-Aldrich Chemical Co. Ltd., St. Louis, MO, USA) and subsequently transcribed into cDNA. A qPCR was performed to assess the expression levels of various genes, according to the procedures as previously described [18]. Quantitative analyses were conducted using the 2^−ΔΔCT^ method, and all the data were normalized to the GADPH expression. The primer sequences used were as follows: TNF–α: 5′-CAGGCGGTGCCTATGTCTC-3′ and 5′-CGATCACCCCGAAGTTCAGTAG-3′; IL-1β: 5′-GCAACTGTTCCTGAACTCAACT-3′ and 5′-ATCTTTTGGGGTCCGTCAACT-3′; IL–6: 5′-TCTATACCACTTCACAAGTCGGA-3′ and 5′-GAATTGCCATTGCACAACTCTTT-3′; Caspase-3: 5′-CTGGACTGTGGCATTGAGAC-3′ and 5′-GCAAAGGGACTGGATGAACC-3′; Bax: 5′-CCGGCGAATTGGAGATGAACT-3′ and 5′-CCAGCCCATGATGGTTCTGAT-3′; Bcl-2: 5′-TCAGAGCGAGAAGGTAGGGA3′ and 5′-CTGTGGGGTAACAAGAAGGTC-3′; GAPDH: 5′-AGAAACCTGCCAAGTATGATGAC-3′ and 5′-AGAAACCTGCCAAGTATGATGAC-3′; CXCL10: 5′-TGAATCCGGAATCTAAGACCATCAA-3′ and 5′-AGGACTAGCCATCCACTGGGTAAAG-3′; CSF3: 5′-AAGCTGGTGAGTGAGTGTGC-3′ and 5′-GGCCATTCCCAGTTCCA-3′; CCL2: 5′-AGCAGCAGGTGTCCCAAAGA-3′ and 5′-GATCTCATTTGGTTCCGATCCA-3′; and CCL5: 5′-CCTGCTGCTTTGCCTACATTGC-3′ and 5′-ACACACTTGGCGGTTCTTTCGG-3′. 

### 2.12. Determination of SOD, GSH, and MDA Content

The RAW264.7 macrophages were digested, centrifuged, and resuspended. The cell concentration was adjusted to 1 × 10^6^ cells/mL and then inoculated into a 6-well plate with 2 mL per well. In the experimental group, 50 µL of bacterial suspension was added to each well (see the treatment of the *L. plantarum* A106 in Section 2.5). The control group only used the complete medium. The LPS was added at a final concentration of 1 µg/mL to the experimental group 4 h before the end of the cultivation. The cells were lysed using a cell lysis buffer (Beyotime) and then collected and centrifuged, and the supernatant was taken for the determination of the SOD, GSH, and MDA contents using a total SOD assay kit with WST-8, a GSH assay kit with DTNB, and an MDA assay kit (Beyotime), according to the instructions of each kit.

### 2.13. Determination of ROS Level

The cells cultivated in the 6-well plate underwent the same treatment as described in Section 2.12. The levels of ROS were analyzed with a ROS assay kit (Beyotime) following the provided instructions. The cells were collected, centrifuged, and resuspended before the levels of ROS were analyzed using flow cytometry (CytoFLEX, Beckman Coulter, Indiana, IN, USA).

### 2.14. Determination of Mitochondrial Membrane Potential (MMP)

The evaluation of the MMP is an important indicator to assess the normal function of the mitochondria. The MMP was measured using an enhanced mitochondrial membrane potential assay kit with JC-1 according to the instructions of operation (Beyotime). Firstly, the cellular states and staining conditions were observed under the fluorescence microscope. Then, the cells were collected, centrifuged, and resuspended, and the intensity of the fluorescence was assayed through a flow cytometer (Beckman Coulter, CytoFLEX).

### 2.15. Apoptosis Assay

Generally, the loss of MPP is a sign of cellular apoptosis. In this study, cellular apoptosis was also determined using an Annexin V-FITC apoptosis detection kit, according to the protocol of the manufacturer (Beyotime). Briefly, the cells were seeded in a 6-well plate, then collected, centrifuged, and resuspended in 195 μL of Annexin V-FITC binding buffer at the end of the different treatments. This was followed by the addition of 5 μL of Annexin V-FITC and 10 μL of PI (propidium iodide) staining solution, before incubation at room temperature for 10–20 min in the dark and subsequent storage in an ice water bath for analysis in a flow cytometer.

### 2.16. Statistical Analysis

The statistical analysis was conducted using Origen 2019b and SPSS Statistics 25 software using a one-way ANOVA, where *p* < 0.05 was considered as statistical significance. All the experiments were expressed as the mean ± SEM.

## 3. Results

### 3.1. Effect of Culture Supernatant, Bacterial Cells, and Cell-Free Extracts on the Scavenging Rate of Different Radicals In Vitro

**Figure 1:**
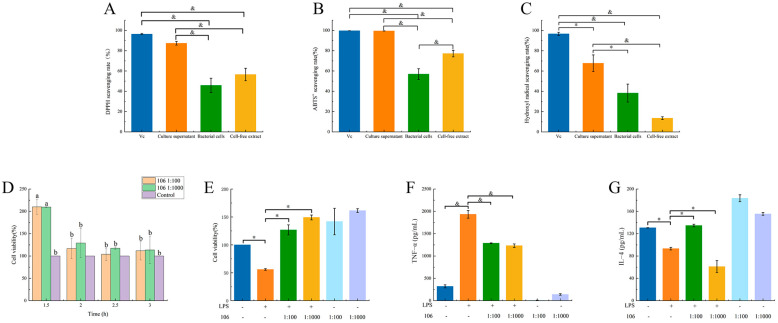
Scavenging rates of DPPH (**A**), ABTS+ (**B**), and hydroxyl radicals (**C**) of different fractions derived from *L. plantarum* A106 (**D**) Changes of cell viability depending on different treatment time (**E**). The effect of *L. plantarum* A106 treatment on the cell viability of LPS-induced RAW264.7 macrophages; and the effect of *L. plantarum* A106 treatment on the levels of TNF–α (**F**) and IL–4 (**G**) in LPS-induced RAW264.7 macrophages. The data are expressed as the mean ± SEM; and the *p* value was determined by a one-way ANOVA followed by multiple comparisons. * denotes a significant difference at *p* < 0.05, and & denotes a significant difference at *p* < 0.01. Different letters indicated significant differences (*p* < 0.05).

The supernatant, bacterial cells, and cell-free extracts of the *L. plantarum* A106 culture were prepared separately and tested for the scavenging rates of DPPH, ABTS^+^ and hydroxyl radicals. It can be seen from Figure 1 that the DPPH scavenging rate of the *L. plantarum* A106 culture supernatant was significantly higher than those of the bacterial suspension and the cell-free extract group (*p* < 0.01). Furthermore, the DPPH scavenging rate of the bacterial suspension was also obviously higher than that of the cell-free extract group (*p* < 0.01). Vitamin C (Vc) was the positive group (Figure 1A). Moreover, the results of the ABTS^+^ radical scavenging rate also indicated a similar trend to the DPPH scavenging rate. (Figure 1B). Interestingly, the hydroxyl radical scavenging rate showed the same trend as the DPPH, with the only difference being that the cell-free extract group had a higher hydroxyl radical scavenging rate than the bacterial suspension group (Figure 1C).

### 3.2. Effect of L. plantarum A106 on the Cell Viability of LPS-Induced RAW264.7 Macrophages

The cell viability was determined with the CCK-8 assay kit. As shown in Figure 1D, the *L. plantarum* A106 demonstrated no cytotoxicity towards RAW264.7 macrophages. Additionally, the cell viability of the *L. plantarum* A106-treated group (1:100 or 1:1000) was significantly higher (*p* < 0.05) than that of the control group at 1.5 h. Furthermore, there were no significant differences among all groups after cultivating for 2 h; as a result, 2 h was determined to be the ideal duration for the CCK-8 treatment. After a 2 h CCK-8 treatment, there was a noteworthy reduction in the cell viability of the LPS-treated group when compared to the control group. However, the treatment with *L. plantarum* A106 (1:100 or 1:1000) significantly impeded the decrease in cell viability (*p* < 0.05) (Figure 1E).

### 3.3. Effect of L. plantarum A106 Treatment on the LPS-Induced Secretion of Inflammatory Factors

As shown in Figure 1F,G, compared with the control group, the levels of the pro-inflammatory factor TNF–α were significantly increased (*p* < 0.01) and of the anti-inflammatory factor IL–4 were significantly reduced in the RAW264.7 macrophages that were stimulated by LPS (*p* < 0.05). In contrast, the treatment with the *L. plantarum* A106 significantly reduced the TNF–α levels (*p* < 0.01) but obviously increased the IL–4 levels (*p* < 0.05).

### 3.4. Selection and Functional Enrichment Analysis of DEGs

**Figure 2:**
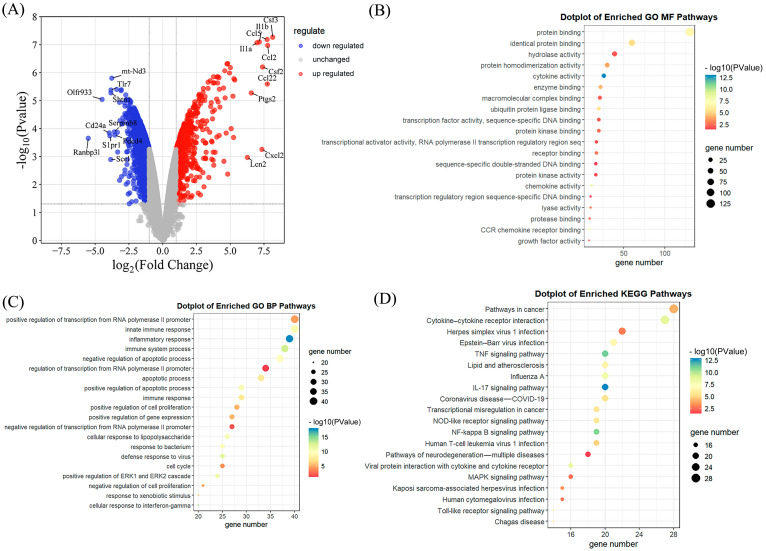
DEGs and enrichment analysis. (**A**) Volcano plot showing DEGs in the LPS-treated group vs. the control group; (**B**) GO:MF enrichment results (top 20); (**C**) GO:BP enrichment results (top 20); and (**D**) KEGG enrichment results (top 20).

Among the 2465 differentially expressed genes (DEGs1) identified from the GSE186886 dataset, 519 genes were upregulated and 1946 genes were downregulated. The selection criteria were *p* < 0.05 and |log2 (fold change, FC)| > 1. All DEGs1 are presented in a volcano plot (Figure 2A), with red indicating the upregulated genes, blue indicating the downregulated genes, and gray representing the genes that did not show significant expression changes in the dataset.

To further investigate the functions of the DEGs, the GO and KEGG analyses were performed on the DEGs2 (differentially expressed genes from DEGs1 with |log2 (fold change, FC)| > 2) using DAVID, and the top 20 enriched entries were visualized using bubble charts. As shown in Figure 2D, the KEGG enrichment analysis presented several pathways that were significantly enriched with inflammatory responses, including the cytokine—cytokine receptor interaction; the TNF signaling pathway and IL–17 signaling pathway; the NOD-like receptor signaling pathway; and the NF-kappa B signaling pathway. As depicted in Figure 2C, the BP (biological process) category was predominantly composed of the innate immune response, the positive regulation of transcription from the RNA polymerase II promoter, the inflammatory response, and the immune system process. The MF (molecular function) category (Figure 2B) comprised protein binding, identical protein binding, hydrolase activity, protein homodimerization activity, and cytokine activity. 

### 3.5. GSEA Analysis of Biological Processes and Functions

**Figure 3:**
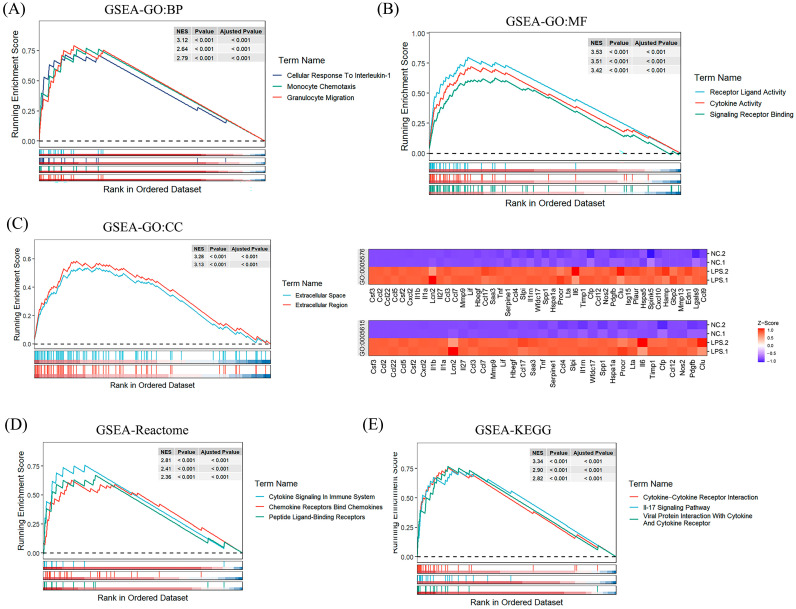
GSEA enrichment analysis. (**A**) The significant GSEA sets for the GO-BP pathways; (**B**) the significant GSEA sets for the GO-MF pathways; (**C**) the significant GSEA sets and heatmap for the GO-CC pathways; (**D**) the significant GSEA sets for the Reactome pathways; and (**E**) the significant GSEA sets for the KEGG pathways.

The gene set enrichment analysis (GSEA) was conducted to delve deeper into the biological functions and pathways associated with DEGs2, because the results of the GO analysis showed that DEGs2 were mainly enriched in extracellular space and the extracellular region (Figure 3C). In terms of biological processes (Figure 3A), the main enrichment was related to the response to IL–1, monocyte chemotaxis, and granulocyte migration. Similarly, at the molecular function level (Figure 3B), there was found to be high enrichment of the cytokine activity, cytokine receptor binding, and chemokine activity. The pathway analysis based on KEGG and Reactome highlighted the role of related pro-inflammatory cytokines (Figure 3D,E). Therefore, these results suggest that the LPS treatment of RAW264.7 macrophages may contribute to the development of inflammatory responses.

### 3.6. Construction of Protein–Protein Interaction Network, Selection of Hub Genes, and Metascape Enrichment Analysis

**Figure 4:**
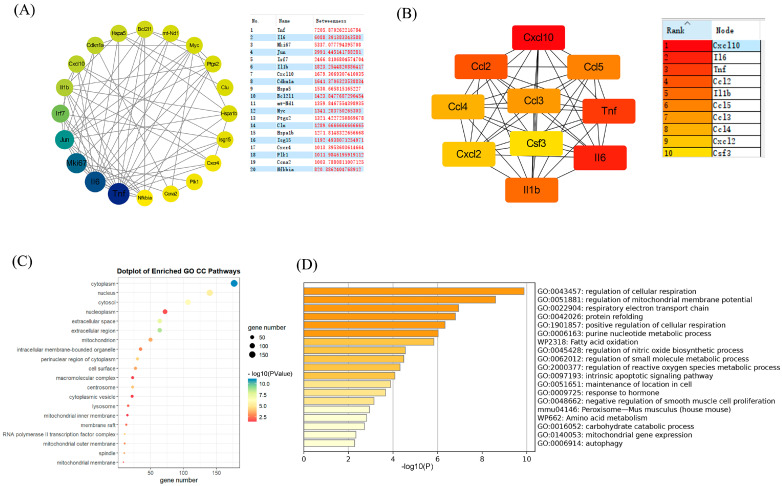
PPI and mitochondrial Metascape analysis. (**A**) Top 20 key genes featured by CytoNCA; (**B**) a key module with 10 genes, the key gene screened by the MCC method; (**C**) the GO:CC enrichment results (top 20); and (**D**) the Metascape results of mitochondrial DEGs.

After uploading the selected DEGs2 to the STRING online database with a confidence threshold of >0.7 and visualizing the results in Cytoscape software (version 3.9.1), any isolated nodes were removed to establish a protein–protein interaction (PPI) network consisting of 164 nodes and 667 edges. Subsequently, the betweenness centrality of the nodes was assessed using the CytoNCA plugin, where darker node colors indicated higher betweenness centrality values (Figure 4A). In addition, the top 10 genes were identified in Cytohubba using the maximal clique centrality (MCC) method (Figure 4B), where the node color represented connectivity (red for higher degrees and yellow for lower degrees). Integrating the outcomes of the betweenness centrality and maximal clique centrality analyses, we propose that TNF, IL–6, Cxcl10, and IL–1β potentially play pivotal roles in modulating LPS-induced infections.

According to the CC (cellular component) analysis (Figure 4C), the above findings were mainly associated with organelles such as the nucleus and mitochondrion. Given that the role of mitochondria in cellular functionality is critical, because they serve as the cell’s powerhouses by producing ATP via respiration and regulating cellular metabolism and apoptosis, we conducted an enrichment analysis on 50 DEGs associated with mitochondria identified in the CC (cellular component) using the Metascape online platform (https://metascape.org). The results primarily indicate enrichment in the regulation of cellular respiration, the regulation of mitochondrial membrane potential, and the respiratory electron transport chain (Figure 4D).

### 3.7. Effect of L. plantarum A106 on Inflammatory-Related Gene Expression Levels 

**Figure 5:**
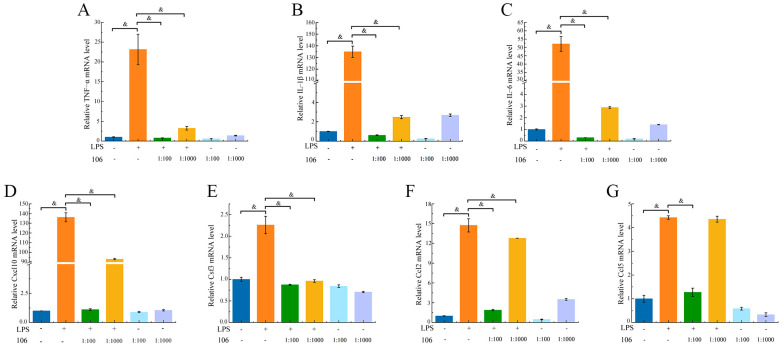
Effect of the *L. plantarum* A106 treatment on the relative levels of the expression of TNF–α mRNA (**A**), IL–1β mRNA (**B**), IL–6 mRNA (**C**), Cxcl10 mRNA (**D**), Csf3 mRNA (**E**), Ccl2 mRNA (**F**), and Ccl5 mRNA (**G**) in the LPS-induced oxidative stress in RAW264.7 macrophages. The data are expressed as the mean ± SEM; and the *p* value was determined by a one-way ANOVA followed by multiple comparisons. & denotes significant difference at *p* < 0.01; and 106 represents *L. plantarum* A106.

TNF–α, IL–1β, IL–6, Cxcl10, Csf3, Ccl2, and Ccl5 were identified as the hub genes and the significantly upregulated genes. As demonstrated in Figure 5, the expression of TNF–α, IL–1β, IL–6, Cxcl10, Csf3, Ccl2, and Ccl5 genes increased following the LPS stimulation (*p* < 0.05). Compared to the LPS-treated group, the addition of *L. plantarum* A106 significantly downregulated the expression of TNF–α, IL–1β, IL–6, Cxcl10, Csf3, Ccl2, and Ccl5 genes, indicating that *L. plantarum* A106 can mitigate the inflammatory levels caused by LPS treatment of RAW264.7 macrophages and exert anti-inflammatory effects.

### 3.8. Effect of L. plantarum A106 on LPS-Induced SOD, GSH, and MDA Activities

**Figure 6:**
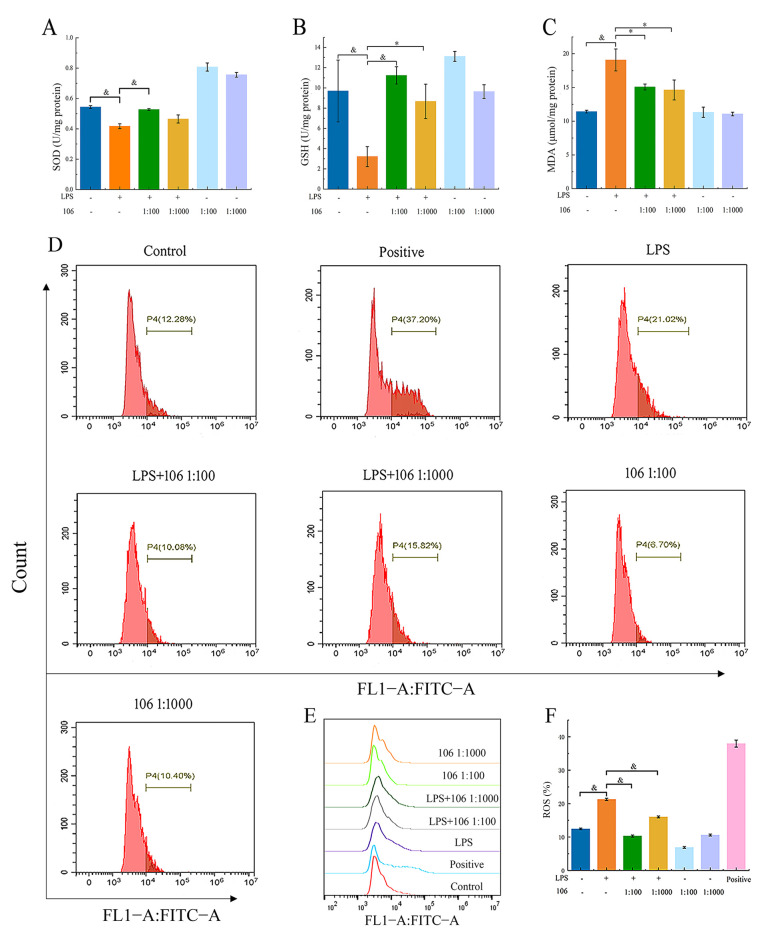
Effect of *L. plantarum* A106 treatment on the SOD (**A**), GSH (**B**), and MDA (**C**) activity and the cellular ROS levels of the LPS-induced oxidative stress in RAW264.7 macrophages; (**D**) ROS levels analyzed by flow cytometry; (**E**) overview of ROS levels in different groups; and (**F**) comparative histogram of the ROS levels in different groups. The data are expressed as the mean ± SEM; and the *p* value was determined by a one-way ANOVA followed by multiple comparisons. * denotes a significant difference at *p* < 0.05; & denotes a significant difference at *p* < 0.01; and 106 represents *L. plantarum* A106.

RAW264.7 macrophages were used to establish a LPS-induced oxidative stress model to further explore the antioxidant capacity of *L. plantarum* A 106. The results are shown in Figure 6. Under the stimulation of LPS, the SOD and GSH viability of RAW264.7 macrophages was significantly reduced, and the MDA viability was significantly increased compared to the control (*p* < 0.01) (Figure 6A–C). However, the addition of *L. plantarum* A106 significantly increased the SOD and GSH viability (*p* < 0.01) and significantly reduced the MDA viability (*p* < 0.05). The above results indicated that treatment with *L. plantarum* A106 might slow down the LPS-induced oxidative stress in RAW264.7 macrophages.

### 3.9. Effect of L. plantarum A106 on LPS-Induced ROS Levels

The results of the ROS levels are shown in Figure 6D. The ROS level was significantly elevated in the RAW264.7 macrophages stimulated by LPS compared to the control group, while the addition of *L. plantarum* A106 significantly reduced the ROS levels (*p* < 0.01), and the high dose group (1:100) was more effective than the low dose (1:1000). This result suggests that *L. plantarum* A106 had the potential to effectively reverse the increase in the ROS levels induced by LPS and alleviate the oxidative damage in LPS-induced RAW264.7 macrophages.

### 3.10. Effects of L. plantarum A106 on LPS-Induced Mitochondrial Membrane Potential (MMP)

**Figure 7:**
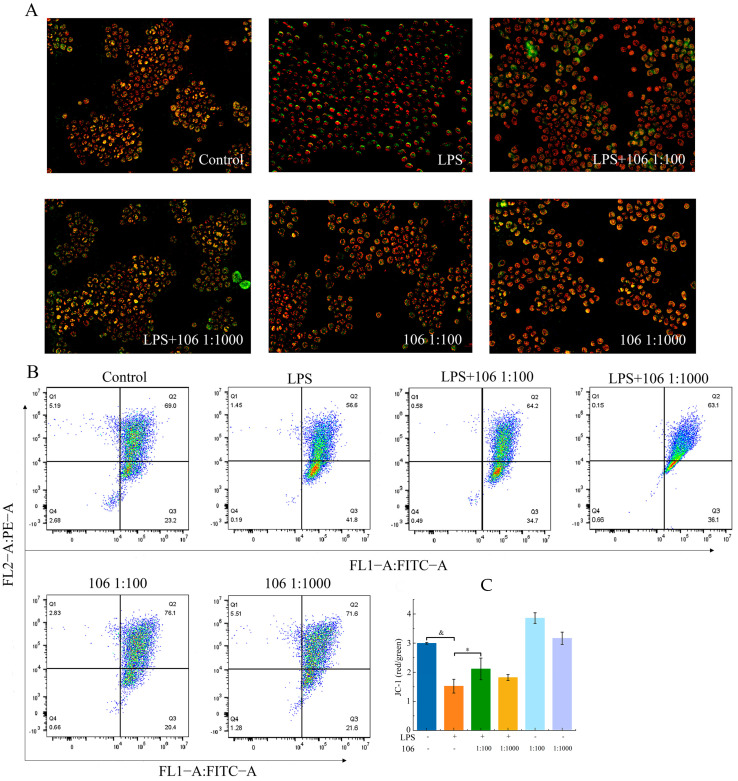
Effect of *L. plantarum* A106 treatment on the mitochondrial membrane potential of LPS-induced oxidative stress in RAW264.7 macrophages. (**A**) Changes of mitochondrial membrane potential in different groups under the fluorescence microscope; (**B**) the flow cytometric analysis of the mitochondrial membrane potential in different groups; and (**C**) a comparative histogram of the levels of mitochondrial membrane potential in different groups. The data are expressed as the mean ± SEM; and the *p* value was determined by a one-way ANOVA followed by multiple comparisons. * denotes a significant difference at *p* < 0.05; & denotes a significant difference at *p* < 0.01; and 106 represents *L. plantarum* A106.

The above analysis revealed that lipopolysaccharide (LPS) might influence the mitochondrial membrane potential (Figure 4D). Therefore, fluorescence microscopy (Olympus, 50X) was used to preliminarily observe the cells in each treatment group. As shown in Figure 7A, more cells were with green fluorescence in the LPS-induced RAW264.7 macrophages compared to the control group, indicating a loss of MMP; subsequently, the green fluorescence weakened visually after treatment with the *L. plantarum* A106, but it was difficult to calculate the exact ratio between the red and green fluorescence. Therefore, flow cytometry was employed to detect the precise fluorescence change. As shown in Figure 7B,C, the red/green fluorescence intensity ratio obviously decreased in the LPS-induced RAW264.7 macrophages (*p* < 0.01) but remarkably increased after the treatment with *L. plantarum* A106 (*p* < 0.05).

### 3.11. Effects of L. plantarum A106 on LPS-Induced Apoptosis

**Figure 8:**
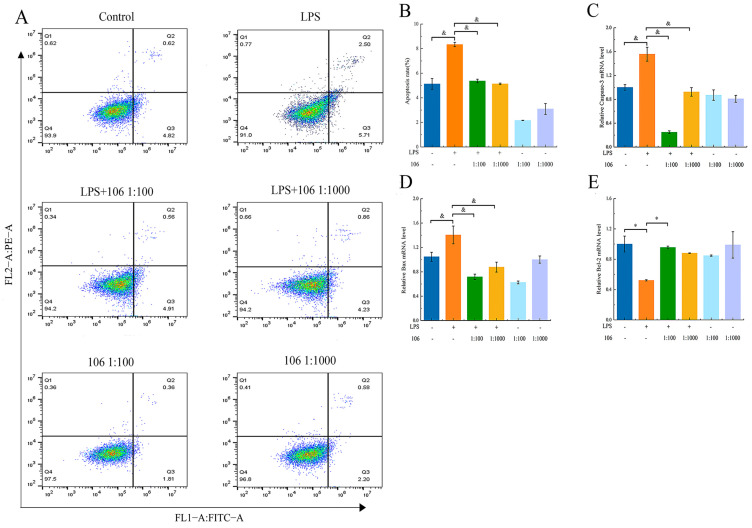
Effect of *L. plantarum* A106 treatment on cell apoptosis and apoptosis-related gene expression in LPS-induced oxidative stress in RAW264.7 macrophages. (**A**) Flow cytometry analysis of apoptosis in different groups; (**B**) combined analysis of the flow cytometry results among different groups; (**C**) Caspase–3 mRNA expression level; (**D**) Bax mRNA expression level; and (**E**) Bcl–2 mRNA expression level in LPS-induced oxidative stress in RAW264.7 macrophages. The data are expressed as the mean ± SEM; the *p* value was determined by a one-way ANOVA followed by multiple comparisons; and & denotes a significant difference at *p* < 0.01. * denotes a significant difference at *p* < 0.05.

The loss of mitochondrial membrane potential is often a precursor to apoptosis, leading to the activation of downstream caspases and resulting in cell apoptosis. In this study, flow cytometry was used to detect the apoptotic status of the cells in each treatment group. The results, as shown in Figure 8A,B, indicate that stimulation with LPS significantly increased the percentage of apoptotic cells compared to the control, and the addition of *L. plantarum* A106 significantly decreased the percentage of apoptotic cells (*p* < 0.01). Furthermore, there was no significant difference between the LPS-induced group with a low dose of *L. plantarum* A106 and with a high dose of *L. plantarum* A106. 

Additionally, we assessed the impact of various treatments on the expression of apoptosis-related genes (Caspase–3, Bcl–2, and Bax) through a qPCR. As shown in Figure 8C,D, compared with the control group, the Bcl–2 gene expression was significantly reduced (*p* < 0.01), but the Caspase–3 and Bax gene expressions were significantly increased in the RAW264.7 macrophages stimulated by LPS (*p* < 0.01), while the addition of *L. plantarum* A106 significantly upregulated the Bcl–2 expression (*p* < 0.01) but significantly downregulated the Caspase-3 and Bax expressions (*p* < 0.01).

## 4. Discussion

More and more studies have provided reliable evidence for the biological activities of LAB, identifying their antioxidant, antitumor, and immunomodulatory properties. LAB have also been considered as a potential natural antioxidant [19,20]. However, there has been the lack of a comprehensive and precise research method to deeply explore the anti-inflammatory and antioxidative effects of LAB. Therefore, this study combined bioinformatics analysis and experiment validation to investigate the potential anti-inflammatory and antioxidative effects of *L. plantarum* A106 and its underlying mechanisms.

DPPH, ABTS^+^, and hydroxyl radicals are electronic reduction products of a class of oxygen in organisms and common reactive oxygen species. Thus, the antioxidant activity of LAB can be measured with high ratios of DPPH, ABTS^+^, and hydroxyl radical scavenging. Tang et al. (2017) assessed the antioxidant activity exhibited by the fermented product of *L. plantarum* MA2, which was isolated from the traditional kefir grains in Tibet, and demonstrated that the DPPH radical scavenging rates of both the MA2 fermentation supernatant and the intact cells were superior to those of a cell-free extract [21]. Interestingly, this results were similar to our results. Specifically, in this study, *L. plantarum* A106 supernatant or cell-free extracts also had a higher DPPH radical scavenging rate. Moreover, the scavenging rates of the ABTS^+^ and hydroxyl radicals also presented similar results to the DPPH radical rate, indicating that *L. plantarum* A106 has a strong antioxidant activity. 

It has also been reported that LAB have an immunological regulation effect [22,23]. Li et al. (2021) found that *L. plantarum* KSFY06 upregulated the anti-inflammatory factor IL–10 and downregulated the pro-inflammatory factors IL–6 and TNF–α to prevent the inflammatory response of an LPS-induced acute liver injury in mice [24]. Prolonged inflammatory processes increased the ROS production and caused oxidative stress; as a result, anti-inflammation might also mitigate the oxidative damage to cells [25]. In this study, the treatment with *L. plantarum* A106 upregulated the anti-inflammatory factor IL–4 and downregulated the gene expression of the pro-inflammatory factor TNF–α. However, further study was needed to explore the anti-inflammatory and antioxidative mechanisms of *L. plantarum* A106. 

Subsequently, bioinformatics analysis was performed on the GSE186886 dataset, identifying 2465 differentially expressed genes (DEGs), including significantly upregulated inflammatory-related genes Csf3, Ccl5, and Ccl. Further KEGG enrichment analysis of the DEGs2 (genes from DEGs1 with |log2 (fold change, FC)| > 2) revealed key inflammatory pathways including cytokine—cytokine receptor interaction, a TNF signaling pathway, and an IL–17 signaling pathway [26]. The GO BP and GO MF analyses additionally indicated enrichment in the innate immune response, inflammatory response, and cytokine activity [27]. These findings suggest the potential impact of LPS on the inflammatory signaling pathways.

The gene set enrichment analysis (GSEA) results underscored the significant roles of these genes in biological functions and pathways. Specifically, these DEGs were notably enriched in cytokine—cytokine interactions, the IL–17 signaling pathway, and cytokine signaling in the immune system through the KEGG and Reactome pathway analyses, highlighting their importance in inflammatory signal transduction. Moreover, the GO analysis revealed that these genes predominantly clustered in extracellular spaces and regions, as well as in the biological processes and molecular functions associated with the innate immune and inflammatory responses. These findings further unveiled the mechanism behind LPS’s promotion of inflammatory responses in RAW264.7 macrophages. These insights provide a deeper understanding of the LPS-induced inflammatory mechanisms.

By integrating the analyses of the betweenness centrality and maximal clique centrality (MCC) through the use of the Cytoscape software (version 3.9.1), we identified several key hub genes: TNF, IL–6, Cxcl10, and IL–1β [28]. These genes were likely to play critical roles in the inflammatory signaling induced by LPS [29]. Furthermore, the enrichment analysis of the mitochondrial-related differentially expressed genes (DEGs) using Metscape suggested that LPS perturbed cellular respiration, mitochondrial membrane potential, the respiratory electron transport chain, and the process of apoptosis [30]. These findings reveal that while LPS promotes inflammatory responses, it also disrupts the cellular energy metabolism and redox reactions, affecting cellular homeostasis.

TNF and IL–1β are pro-inflammatory cytokines that play pivotal roles in the initiation and perpetuation of inflammation. They were involved in the upregulation of inflammatory pathways, enhancing the production of other cytokines and recruiting immune cells to sites of infection or injury, thereby amplifying the inflammatory response [31]. IL–6 was a multifunctional cytokine that not only facilitated inflammation but also possessed immunoregulatory functions [32]. *L. helveticus* SBT2171 significantly reduced the LPS-induced expression and secretion of IL–6 and IL–1β cytokines in peritoneal macrophages, leading to anti-inflammatory effects [33]. Cxcl10, a chemokine induced by IFN–γ, promoted the migration of immune cells to inflamed tissues during the LPS-induced inflammation, further exacerbating the inflammatory milieu [34]. Consistent with these findings, our bioinformatics analysis also identified these four molecules as hub genes in a macrophage model treated with LPS. Additionally, our qPCR results demonstrated an increase in the expression of these genes following the LPS stimulation. Compared to the LPS-treated group, the addition of *L. plantarum* A106 significantly downregulated the expression of these genes, suggesting that these cytokines may be potential targets for the therapeutic intervention of inflammatory diseases with *L. plantarum* A106.

Through a meta-analysis of mitochondrial-related DEGs, we discovered that LPS may significantly affect cellular respiration and the mitochondrial electron transport chain. Cellular respiration, a crucial process in cellular energy metabolism, is accompanied by the production of reactive oxygen species (ROS), particularly within the mitochondrial electron transport chain. ROS include free radicals such as superoxide anions (O2-) and non-radical forms like hydrogen peroxide (H_2_O_2_) [35]. Excessive ROS can lead to oxidative stress, damaging proteins, lipids, and nucleic acids, thereby triggering various diseases [36]. To counteract the potential harm caused by ROS, cells have developed a complex antioxidant system, comprising enzymes like superoxide dismutase (SOD), catalase, and glutathione peroxidase, as well as non-enzymatic antioxidants such as glutathione and vitamin C [37]. These antioxidants neutralize the ROS and maintain the redox balance within the cell [38]. In this study, we found that the plant-derived *L. plantarum* A106 could reduce the ROS and MDA levels in RAW264.7 macrophages and enhance the SOD and GSH activities, thus boosting the cellular antioxidant capacity and ameliorating the LPS-induced oxidative stress in RAW264.7 macrophages. This result was consistent with the result that *L. plantarum* DP189 significantly reversed the MPTP-induced decrease in SOD and GSH-Px activity, increased the MDA activity, and improved the antioxidant capacity in Parkinsonian mice [3]. 

The bioinformatics analysis suggested that LPS might interfere with the regulation of the mitochondrial membrane potential, a critical indicator of mitochondrial health and function and the driving force behind ATP synthesis via oxidative phosphorylation. Disruption of this potential was often a precursor to mitochondrial apoptosis, with changes triggering the release of pro-apoptotic factors such as cytochrome c, leading to the activation of downstream caspases and resulting in cell apoptosis [39,40]. In our study, we employed JC-1 staining to observe a significant decrease in the mitochondrial membrane potential (MMP) induced by LPS, suggesting an increase in the mitochondrial pathway apoptosis. Subsequently, through flow cytometry and qPCR assays, we detected an increase in the apoptosis rates and an elevated expression of the anti-apoptotic gene Bcl–2, alongside a reduced expression of the pro-apoptotic genes Caspase–3 and Bax following the LPS treatment of RAW264.7 macrophages. The treatment of RAW264.7 macrophages with *L. plantarum* A106 reversed the decline in the MMP and increased the apoptosis rates caused by LPS. This experimental result was consistent with Li et al. (2022b), who reported that dandelion extract reduced the apoptosis rate of RAW264.7 macrophages and attenuated the LPS-induced inflammatory response [41].

## 5. Conclusions

In summary, this study demonstrates that *L. plantarum* A106 possesses anti-inflammatory and antioxidant properties. The bioinformatics analysis and experimental results indicate that TNF, IL–1β, IL–6, and Cxcl10 may be potential targets for *L. plantarum* A106 in exerting its anti-inflammatory role. Additionally, *L. plantarum* A106 can improve the mitochondrial function in RAW264.7 macrophages, enhance the activities of SOD and GSH, and reduce the levels of MDA and ROS, thereby mitigating oxidative stress and reducing LPS-induced cell apoptosis.

## Data Availability

The original contributions presented in the study are included in the article, further inquiries can be directed to the corresponding author.
